# An Introductory Lecture on Anatomy

**Published:** 1870-01

**Authors:** E. Lloyd Howard

**Affiliations:** Professor of Anatomy in the Baltimore College of Dental Surgery.


### ARTICLE II.

### *An Introductory Lecture on Anatomy.*

By E. LLOYD HOWARD, M.D.,

Professor of Anatomy in the Baltimore College of Dental Surgery.

(Delivered to the Class of the Baltimore College of Dental Surgery.)

In commencing the study of any branch of science, it is well to take a broad and general view of its relations, and to clearly define its outlines, before entering into an examination of its various details.

In beginning the study of anatomy then, before proceeding to investigate the structure of the human organism and its component organs, let us first try to get a clear conception of what organs and organisms are, and of their relations to inorganic nature.

The first great division we make in the classification of natural objects, is into living and inanimate; and inasmuch as we find that life is never manifested except in connection with matter built up in a certain definite structure, or *organized* matter, we apply the term *organic* to living nature and *inorganic* to dead nature. The term *organism* is used to denote that combination of parts which constitutes an individual living thing—whether animal or vegetal. An *organ* is one of the constituent parts of the organism. Thus the human body is an *organism;* the brain, the heart, the liver, the muscles, etc., are the organs of which it is composed.

*Anatomy* is that science which treats of the structure of the organism and its organs.

Anatomy is commonly divided into Descriptive, Comparative, General and Microscopical Anatomy,

*Descriptive human anatomy* is the branch to which your attention will chiefly be directed here, but before doing so, let us take a brief survey of the field of comparative anatomy; for there is a grand unity in the creation, and man occupies a peculiar relation to other animals which should not be lost sight of.

In the first place let us look a little more closely into the differences between organic and inorganic nature. The chief point of difference is to be found in the manner of growth; for inorganic matter may be said to grow. The crystal grows, it does more; it grows in a definite manner and form. The various faces and angles are built up in the most orderly and regular manner, according to a fixed plan, and if by accident or design you break off one of the angles of the growing structure, see how it hastens to repair the injury, concentrating, as it were, its forces at that point, and building there chiefly, until the angle is restored and the proper form completed. Moreover, crystals seem to have a somewhat definite size like organisms, a limitation of growth, after which is reached is ceases to grow, but from the remaining material new growths start into existence. Now what is it that gives to each crystal its peculiar shape? Why does one substance take the form of a cube, another of a rhomb, a third of a pyramid? Surely it seems that there is some inherent directive agency here! We call it polarity, but what its essential nature is we know not. It probably depends in great part upon the shape of the atoms—the bricks of which the structure is composed.

But there is one thing about this growth which we must not lose sight of. It is, that the growth is by a system of aggregation. A particle of some foreign matter, as a speck of dust, may be the nucleus about which the process of crystallization begins; upon this the crystal starts, and then growth takes place by the simple deposition of fresh matter layer upon layer, just as you would build a wall with bricks.

Now let us turn to the growth of organized matter, what do we find?

In the first place no foreign matter can here serve as the foundation for our structure; this foundation, *nucleus*, must have come from some pre-existing organism of a type similar to that about to be formed; and the nucleus itself must be organic and living. We have no example of spontaneous generation. It is true the discussion is still being carried on, but at present, the weight of evidence is upon the side of the doctrine that every living thing is descended from a similar pre-existing organism.

The starting point then for each plant and animal must be itself a living thing. When and how the first germ of each type came into existence, science can not tell.

In the second place, the mode of growth of the organism is peculiar. Not only like the crystal has it the directive agency, determining its form, but that form is built up in a peculiar way. It is no longer a simple aggregation of particles—a piling of brick upon brick. The living organism first absorbs within its interior the crude material for the structure, its pabulum—food; and then in some mysterious way, changes this pabulum into another substance, or rather into other substances, which finally become converted into the tissues of the plant or animal.

Now please bear this distinction firmly in your minds for *it* is the most fundamental one. *The inorganic* grows by simple additions of material to its surface. *The organic*, by first absorbing, then assimilating, and then adding the newly formed material to its structure *from within.*

In the lower organisms this process of growth is comparatively simple. Each part of the plant or animal seems endowed with the same powers and functions of the whole. The structure of such organisms is but a granular mass, like a lump of jelly; its food is absorbed by any part of the surface with which it may come into contact; then permeates the structure and becomes assimilated. You may divide with a knife such an organism into two, four, twenty, or any number of parts, and each continues to grow just like the one from which they were separated.

But as we ascend in the scale of life, the organism becomes more complex. Then we find one portion of the individual set apart for the performance of one function, and another for another, and difference of function implies difference of structure. So the scale ascends until we find the individual organism to be composed of several organs, each doing its own work, as in the plant, the roots to absorb the pabulum or food; the vessels to elaborate and diffuse it throughout the system, and the leaves to respire. So finally we get to the animal with still greater number and complexity of organs; the stomach to receive and digest the food; the vessels and heart to absorb and diffuse the fresh material to all parts of the system; the respiratory apparatus to aid in the physical and chemical process necessary to life; the muscular system to give the power of motion; the nervous system to preserve harmony in all the various parts, and to bring the animal into sentient relations with the external world; and, finally, the organs of excretion to remove the worn out material from the system.

We have thus far been speaking of organisms generally, including both the animal and vegetal; the two great kingdoms into which all living things are divided.

Now, although the distinction between animal and vegetal is commonly easy and broad, and we find no difficulty in defining the horse an animal, and the oak a vegetal, yet in their lower orders it is often very hard to say where they properly belong; and there are still not a few which naturalists are unable to properly classify as vegetal or animal. Thus there are animals incapable of voluntary movement to greater extent apparently than some plants; and plants apparently more sensitive than some animals. Neither does the distinction of stomach hold good in all cases, for there are many animals without a stomach proper, and some plants with it. In fact it has been impossible to find any one point as distinctive between the two kingdoms; they so interdigitate that it is only by an aggregate of several characteristics that an organism can be classed in its appropriate kingdom.

Having thus briefly indicated the chief distinctive features between organic and inorganic nature; and between the vegetal and animal, into which the former is naturally divided; let us look a little more closely at man's position and relations in the animal kingdom.

A superficial glance shows many thousands of different classes and varieties of animals; and we are chiefly impressed at such a view with the fact of this great variety. But when we come to look more closely into the matter, we find underlying these, as it were, great similarities, particularly in the internal structure of animals. Many eminent naturalists have devoted themselves to the study of these similarities, and the result has been, that they have been enabled to classify the entire animal creation with a remarkable simplicity and accuracy.

It has been found that all known animals are naturally divided into four great groups; each group being formed upon a separate and distinct plan, and every animal of each group being strictly conformable to that plan.

These four plans or typical forms are, the Radiate, the Mollusk, the Articulate and the Vertebrate.

In the Radiates, as the name implies, the parts are all arranged around a common centre, from which they diverge like the spokes of a wheel. Take as an example this five-rayed starfish. In the centre is the mouth, immediately beneath this is placed the stomach, sending prolongations into each of the rays, around the mouth is a collar of nerve structure, also sending branches into the rays. On this plan although with great modifications, are constructed the Sea-Urchins, Jelly-fishes, Corals and many others.

The second plan embracing Mollusks, is that expressed in the organization of such animals as the Oyster, Snail, etc. This differs from the plan of the Radiates chiefly in this; that every thing is symmetrical on the sides of the body along the longitudinal axis, at one end of which is the mouth. There is greater complexity and differentiation of parts in this type than in the Radiata and it is regarded as indicating a higher grade of life. Now, at first sight, there appears no great similarity of structure in the oyster and snail, yet by a careful dissection it can easily be proven, that they are both formed according to the same plan, the only difference being in the details of the building, and not in the fundamental parts of the structure.

The third plan, the Articulate, has as its most prominent representatives, insects, beetles, crabs, lobsters and worms. The peculiarities of this group are these; They are composed of a number of rings,more or less closely joined together; running through them all is the alimentary canal; within the rings, also, are contained the nervous system, and the heart and circulatory organs; on the sides are the respiratory organs; the limbs, when legs, are on the lower sides of the rings, when wings on the upper. In this group as in the others, there are apparently great dissimilarities—as between the bee and the worm, and the crab, yet, if time allowed, I could show you that they are all constructed upon essentially the same principal.

In the fourth group, the Vertebrates,—embracing Fishes, Reptiles, Birds and Mammals, and at the head of which is man—we find the essential peculiarity to be, that they have a *backbone*, in virtue of which they have a double structure —a double symmetry.

The backbone is composed of a number of pieces joined together end to end, but unlike the articulates which have simple rings thus joined, the vetebrates have a series of *double* rings. Let me try to illustrate this.

Here you see we have an upper arch, the spine, and a lower arch, the ribs, forming two cavities along the length of the animal. In the upper cavity are contained the sensitive organs, the great nerve centres of animal life. In the lower canal are the organs of digestion, circulation, respiration, etc. Surrounding these two bony rings, are the principal muscles and tegmentary coverings. This double symmetry is the same in all Vertebrated animals from fishes to man.

The outlines of these four groups into which animals are naturally divided, have been very hastily, and I fear at many points dimly drawn. To do justice to the subject would require many lectures, rather than a mere portion of one. But if you have caught the general idea, I have endeavored to convey of animal classification, it will suffice for the present. The details you must fill up hereafter.

Remember then that all the animal creation may be resolved into four typical forms of structure,—the Radiate, the Mollusk, the Articulate and the Vertebrate,—the names of each group pretty clearly indicating the structure, except Mollusk,which is derived from " Mollis," soft, and describes a property which is also common to some Radiates. Upon one of these four plans every animal is built. The structure of the various orders of a group differ, but it is simply a *variation*, not an essentially different structure. Impressed by these similarities, naturalists have for a long time been trying to demonstrate the unity of the animal creation in a linear series, and to show a regular gradation from the lowest to the highest forms of life It has hitherto been impossible to construct this hypothetical chain, and probably will continue so, but some late views by Mr. Darwin, bearing upon this subject, are so full of interest and have made such a stir in the scientific world that I cannot refrain from here briefly calling your attention to them.

In accounting for this mixture of similarities and dissimilarities in the organic world, Mr. Darwin dwells strongly on these two factors: In the first place the organism inherits the features of its parents. In the second place, the offspring differs in some features from the parents, which difference may be transmitted to a third generation. Thus you see there are two opposite tendencies in every newly created organism, the one tending to keep it in a straight line, as it were, the other tending to divert it from this line. Let other factors now be brought to bear, as difference of climate etc. and you can readily imagine that in the millions of births, and during probably millions of years, these struggles with opposite tendencies might have twisted the original image into many different varieties. This we see, to some extent now taking place. Notice for instance, the vast differences which can be produced in certain plants and vegetables by cultivation! See the great differences in horses, sheep and other of our domestic animals produced by a judicious breeding. Take, for example the dog, what great differences between the Newfoundland, the pointer the bull-dog and the terrier, and yet all of these are descended from the same stock! If these changes occur under our sight, what greater changes can occur in the lapse of thousands of years! And so Mr. Darwin thinks that by natural causes operating upon plants and animals, most, if not all of the different species have been formed from a few, if not one, original type. I cannot follow this part of the subject further, but you will find it worthy of your attention, when you have less of the immediately practical to deal with than you have now.

Leaving the three inferior groups let us look a little more closely at the vertebrate skeleton.

We have seen that all vertebrated animals are constructed upon the plan of a series of essentially similar segments, succeeding each other in the axis of the body. In regarding the varieties of this plan it is well to have a standard with which to compare them, and I here present to you what is called a *typical vertebra*, not that you will find such a vertebra actually existing in any animal, for as all animals are variations from the type, you will find their skeletons more or less differing from the pattern. This piece marked C. is intended to represent the body of the vertebra, above and below we have the two rings or arches N. and H. The upper ring N. or neural arch, represents the spinal canal, containing the nerve centres of the spinal cord. The lower ring, H., or haemal arch, represents the cavity in which are contained the organs of digestion, circulation, respiration, etc. Around this framework of double rings are placed the principal muscles and the tegmentary covering.

A series of such structures joined together in the same straight line gives the simplest idea of a typical vertebrate skeleton.

Each of the classes of vertebrates, Fishes, Reptiles, Birds, and Mammals has its peculiar modification of this plan, and so their respective sub-classes have their respective sub-modifications. We will not further consider any of these but the mammalian.

This figure represents a typical mammalian vertebra, which you see is essentially the same thing as the archetype.

You see the spinal canal has become much lessened in proportion to the haemal canal, notice too the process jutting out from the neural arch. But in spite of these changes, you must recognize that the fundamental characters are unaltered. Now I have said that a series of such segments joined together so as to form two hollow cylinders gives the idea of a vertebrate skeleton and that all such skeletons can be reduced to this simple form. But you may say, yes, I can conceive such a skeleton for an animal, but it supposes that the animal is like a snake without a head, and without limbs, how do you account for these developments which most all vertebrates have? Well in answer to that question, I must tell you that the bones of the head are but modified vertebræ, and that the limbs are but offshoots from them. Had we the time to enter fully into the question, I could clearly demonstrate this to you, I could prove to you that the fin of a fish is homologous to the arm of a man, by which I mean that they are essentially the same thing, and bear similar relations to the vertebræ of which they are offshoots, they are constructed upon the same plan, the parts of which they are composed are combined in the same way and almost in the same number.

To recapitulate briefly what we have gone over:

In the first place we have seen that living things differ from inanimate nature chiefly in their mode of growth and development. That organic nature is divided into two great kingdoms—the vegetal and animal. That the animal again is divided into four elementary groups, each group being sub-divided into classes, species, and varieties. That the highest of the elementary groups is the vertebrate. That the highest class of vertebrates is the mammal, and the highest order of mammals is man.

We find man then at the head of created beings according to his physical structure; of course when you take into consideration his intellectual and moral qualites he is immeasureably above all. To examine his structure is the object of anatomy, and in the study of the various organs of which he is composed; their mutual relation and dependence; the most admirable adjustments of means to the end you will find a subject well worthy of your closest attention and if rightly pursued, filled with the most interesting of facts. Anatomy is the very groundwork of your science and art. Without clear ideas of the structure of the tissues how can you expect to work properly upon them? And do not think it will only be necessary for you to pay attention to those organs and tissues with which your art has chiefly to do, for you must remember, that the different organs are so connected, and have such close sympathies with each other that some knowledge of all is necessary to rightly understand the workings of any one.

Moreover, it is proper, that, in aspiring to the high honors which your speciality of the science of Medicine and Surgery may confer upon you, you should be cultivated in this branch about which all scientific men are supposed to have some knowledge.

---

